# γ-Valerolactone Reprograms Tomato Metabolism to Enhance Seedling Growth

**DOI:** 10.3390/metabo16090621

**Published:** 2026-08-27

**Authors:** Xin Tao, Shangbo Yan, Ranran Chen, Fangfang Ren, Hongjie Yang, Wenheng Long, Nan Gao

**Affiliations:** 1 School of Biotechnology and Pharmaceutical Engineering, Nanjing Tech University, Nanjing 211816, China; 2 Key Laboratory of Microbial Biostimulants, Ministry of Agriculture and Rural Affairs, Nanjing Shineking Biotech Co., Ltd., Nanjing 210044, China; 3 Yunnan Yuntianhua Co., Ltd., Kunming 650228, China

**Keywords:** biostimulant, γ-valerolactone (GVL), *Solanum lycopersicum* L., tomato seedlings, widely targeted metabolomics, metabolic reprogramming, KEGG pathway enrichment

## Abstract

**Background:** γ-Valerolactone (GVL), a green volatile platform compound derived from lignocellulosic biomass, has recently been identified as one volatile organic compound produced by the plant growth-promoting rhizobacterium *Stutzerimonas stutzeri* NRCB010. Although our previous transcriptomic and phenotypic studies have verified that exogenous GVL can promote the growth of tomato seedlings, the global metabolic reprogramming behind this promoting effect remains unclear. **Methods:** Tomato seedlings were subjected to exogenous GVL treatments at three concentrations (0, 0.25, 0.5 g/L) for 24 h and 48 h separately. Combined with physiological growth measurements, ultra-high-performance liquid chromatography–tandem mass spectrometry-based widely targeted metabolomics was applied to systematically characterize GVL-mediated metabolic reprogramming. **Results:** GVL remarkably improved seedling height, biomass and root development, with 0.25 g/L GVL showing the strongest promoting effect. A total of 703 metabolites including lipids, flavonoids and alkaloids were identified. Principal component analysis and orthogonal partial least squares discriminant analysis revealed distinct metabolic separation between control and treated groups, verifying time and concentration dependent metabolic shifts. After 24 h, GVL activated primary pathways (the tricarboxylic acid cycle, amino acid and purine metabolism) to supply growth energy; after 48 h, central carbon and secondary biosynthetic pathways were enriched, and 0.5 g/L GVL specifically triggered defensive isoquinoline and indole alkaloid biosynthesis. **Conclusions:** GVL coordinately remodels primary and secondary metabolic networks to accelerate tomato seedling growth, which provides sufficient metabolomic evidence for developing GVL as a novel bio-based plant biostimulant.

## 1. Introduction

Against the backdrop of continuous population growth and climate change, sustainably enhancing crop yield and quality represents a critical challenge for modern agriculture. Although chemical fertilizers have been widely applied to boost crop productivity [1], their overuse poses considerable risks to human health, environmental health and food security [2,3]. Consequently, plant biostimulants derived from microbial resources have emerged as a prominent research focus. Over the past decade, these agents have transitioned from the periphery to the forefront of global strategies for sustainable crop production [4]. Biostimulants are defined as substances or microorganisms that, upon application to plants or the rhizosphere, enhance nutrient uptake [5], improve tolerance to abiotic stresses [6], and increase crop yield and quality by stimulating intrinsic plant physiological processes, without providing direct nutritional supply. This group encompasses humic substances, seaweed extracts, protein hydrolysates, beneficial microorganisms, and butenolide compounds [7].

Among these, lactone-derived molecules have been recognized as promising biostimulants. For instance, exogenous strigolactones improve drought tolerance in tobacco seedlings under varying drought stress intensities and facilitate the growth of flue-cured tobacco (a major cured tobacco type widely used in cigarette production) [8]. While synthetic strigolactone anolog GR24 significantly enhances photosynthetic performance, antioxidant enzyme activities, and biomass accumulation in ornamental sunflower (*Helianthus annuus* cv. Vincent’s Choice), thereby stimulating plant growth and strengthening salt resistance [9]. GR24 effectively alleviates the adverse effects of salt stress in rice by modulating antioxidant enzyme activity and endogenous hormone homeostasis [10]. Karrikin 1 (KAR1) markedly promotes lettuce seed germination by reducing abscisic acid concentrations and elevating hydrolase activity [11]. In addition, KAR1 enhances salt tolerance in creeping bentgrass (*Agrostis stolonifera*) via regulating metabolic reprogramming of specific amino acids, as well as the α-linolenic acid and phenylpropanoid biosynthesis pathways [12].

γ-Valerolactone (GVL) is a versatile platform molecule derived from lignocellulosic biomass via hydrolysis, dehydration, hydrogenation and other processes. Owing to its excellent solvent properties, low toxicity and biodegradability, GVL has attracted extensive attention in the field of green chemistry [13,14]. In our previous work, we identified GVL as a volatile organic compound emitted by plant growth-promoting rhizobacterium (PGPR) *Stutzerimonas stutzeri* NRCB010, and verified its ability to promote tomato seedling growth under both axenic, hydroponic and greenhouse pot conditions [15,16,17]. Rhizosphere microbiome analyses revealed increased relative abundances of *Sphingomonas*, *Tumebacillus* and *Gemmatimonas* at the genus level, implying that GVL may facilitate the assembly of rhizosphere communities associated with plant growth and nutrient transformation. While transcriptomic profiling indicated that GVL triggers extensive reprogramming in tomato seedlings related to hormone signaling, redox metabolism, nucleotide biosynthesis, and defense responses [17]. Nevertheless, systematic investigations dissecting the molecular mechanisms by which GVL regulates plant growth at the metabolome-wide level remain scarce.

Tomato (*Solanum lycopersicum* L.) serves as both a globally important horticultural crop and a model species for studying plant growth and metabolism [18,19]. China is one of the world’s largest tomato producers, contributing 25.78% of the world’s tomato production with 17.81% of the world’s tomato planting area [20].The seedling stage constitutes a critical developmental window for tomato morphogenesis and resistance establishment, and subtle shifts in endogenous metabolism during this period exert profound impacts on subsequent vegetative and reproductive growth. High-sensitivity metabolomics approaches enabling comprehensive profiling of seedling metabolic fingerprints have become a powerful strategy to unravel the mechanisms underlying diverse exogenous stimuli [21,22]. In tomato, metabolomics has been successfully applied to characterize metabolic reprogramming associated with plant development [23], fruit ripening [24,25], drought stress [26], chilling injury [27], and insect resistance [28].

Despite evidence of GVL’s growth-promoting effects, most studies to date are limited to phenotypic and transcriptomic analyses, leaving the remodeling of metabolic pathways insufficiently understood. This study aimed to systematically elucidate the global metabolic reprogramming underlying GVL-mediated tomato seedling growth enhancement using a widely targeted ultra-high-performance liquid chromatography–tandem mass spectrometry (UHPLC-MS/MS)-based metabolomic approach. Multivariate statistical analyses, differential metabolite screening, and Kyoto Encyclopedia of Genes and Genomes (KEGG) pathway enrichment were performed to elucidate the metabolic mechanisms underpinning GVL-mediated growth enhancement. Given its renewable origin and favorable environmental profile, GVL represents a promising candidate for next-generation, bio-based plant growth regulators. This work provides a mechanistic foundation for its future agricultural application.

## 2. Materials and Methods

### 2.1. Plant Material and Experimental Design

Soil was collected from a long-term vegetable cultivation field in Yixing, Jiangsu Province, China (31°12′23″ N, 119°52′89″ E). After removal of debris, the soil was air-dried at room temperature, passed through a 2 mm sieve, and homogenized before use. Its physicochemical characteristics were as follows: 4.25 g/kg organic matter, 49.84 mg/kg nitrate nitrogen, 117.5 mg/kg available P, and a pH of 5.91 [15]. Tomato (*Solanum lycopersicum* L. cv. Xinzhongshusihao) seeds were purchased from Xingke Seed Co., Ltd. (Tianjin, China). Seeds were surface-sterilized to eliminate seed-borne pathogens and ensure uniform initial microbial status across all treatments, and then sown in a plastic tray (54 cm × 28 cm) filled with the prepared soil. When the seedlings developed one pair of true leaves (approximately 9 days after sowing), uniform seedlings were selected and transplanted into 360 mL paper pot containing 320 g of soil, with two seedlings per pot. Each pot was regarded as one independent experimental unit. The experiment was carried out in a greenhouse at Nanjing Tech University (Nanjing, China). The greenhouse was controlled at a constant temperature of 25 ± 2 °C and 70% relative humidity, with a 14 h light/10 h dark photoperiod [29]. For growth-related measurements, data from the two seedlings within the same pot were averaged prior to statistical analysis. The experiment was arranged in a completely randomized design with six replicate pots per treatment. At 2 and 7 days after transplantation, 10 mL of GVL solution was applied as a soil drench by slowly pouring the solution onto the soil surface around the base of each seedling, allowing it to infiltrate the root zone. GVL (purity ≥ 98%) was obtained from Aladdin Biochemical Technology Co., Ltd. (Shanghai, China). GVL (≥98% purity, LR grade) was purchased from Aladdin Biochemical Technology Co., Ltd. (Shanghai, China) and, after dissolving in deionized water, applied as three treatments: 0 (control), 0.25, and 0.5 g/L. The control group received an equal volume of deionized water without GVL.

### 2.2. Plant Growth and Morphological Trait Measurements

Tomato seedlings were harvested at 25 days after transplanting, and growth parameters were quantified using established protocols [30]. Plant height was determined using a standard ruler, and stem diameter was measured using a digital Vernier caliper. Shoots and roots were separated and dried at 60 °C to a constant weight to obtain dry biomass. Root morphological parameters, including total length, surface area and number of root tips were analyzed using an LA-S Plant Root Analysis system (version 2.6.4.2; Wanshen Technology, Hangzhou, China).

### 2.3. Determination of Plant Mineral Nutrient Contents

Dried leaf samples were finely ground and passed through a 0.425 mm sieve prior to analysis. Total nitrogen (N) was determined according to the Chinese agricultural standard (NY/T 2017-2011) [31]. Phosphorus (P), potassium (K), calcium (Ca), and magnesium (Mg) concentrations were quantified by inductively coupled plasma optical emission spectrometry (ICP-OES, iCAP 7400, Thermo Fisher Scientific, Waltham, MA, USA) following the Chinese National Standard (GB/T 35871-2018) [32].

### 2.4. Plant Sample Collection and Metabolite Extraction

Approximately 0.5 g tomato seedling leaf samples were harvested at 24 h and 48 h after the first GVL application, immediately frozen in liquid nitrogen, and stored at −80 °C until analysis. For metabolite extraction, an appropriate amount of the sample solution was transferred to a 2 mL centrifuge tube, followed by the addition of 600 μL pre-chilled methanol containing 2-chloro-L-phenylalanine (4 μg/mL) as an internal standard. Samples were vortexed for 30 s to ensure thorough mixing. Stainless steel grinding beads were added to each tube, and tissues were homogenized using a cryogenic tissue grinder at 50 Hz (three cycles of 60 s each), with intermittent cooling on ice to minimize thermal degradation. The homogenates were ultrasonicated at room temperature for 15 min, with gentle manual shaking every 5 min to maintain dispersion. After centrifugation at 4 °C (12,000× *g*, 10 min), 800 μL of the supernatant was precisely pipetted and filtered twice through a 0.22 μm nylon membrane. The initial filtrate was discarded, and the final filtrate was collected into an LC-MS sample vial [33,34].

### 2.5. Liquid Chromatography (LC) Conditions

Chromatographic separation was performed on a Vanquish ultra-high-performance liquid chromatography (UHPLC) system (Thermo Fisher Scientific, Waltham, MA, USA) equipped with an ACQUITY UPLC^®^ HSS T3 column (2.1 × 100 mm, 1.8 µm; Waters, Milford, MA, USA). The column oven was maintained at 40 °C, and the flow rate was set at 0.3 mL/min with an injection volume of 2 µL. For LC-ESI (+)-MS analysis, the mobile phases consisted of (B2) 0.1% formic acid in acetonitrile (*v*/*v*) and (A2) 0.1% formic acid in water (*v*/*v*). Separation was conducted under the following gradient: 0~1 min, 10% B2; 1~5 min, 10~98% B2; 5~6.5 min, 98% B2; 6.5~6.6 min, 98~10% B2; 6.6~8 min, 10% B2. For LC-ESI (−)-MS analysis, the separation was performed using (B3) acetonitrile and (A3) ammonium formate (5 mM). Separation was conducted under the following gradient: 0~1 min, 10% B3; 1~5 min, 10~98% B3; 5~6.5 min, 98% B3; 6.5~6.6 min, 98~10% B3; 6.6~8 min, 10% B3 [35].

### 2.6. Mass Spectrum Condition

Mass spectrometric detection was performed on Orbitrap Exploris^TM^ 120 mass spectrometer (Thermo Fisher Scientific, Waltham, MA, USA) equipped with an electrospray ionization ion (ESI ion) source. Data were acquired in Full MS-ddMS^2^ (data-dependent MS/MS) mode, allowing simultaneous collection of MS1 and MS/MS spectra. The parameters were as follows: sheath gas pressure, 40 arb; aux gas flow, 10 arb; spray voltages, 3.50 kV (ESI^+^) and −2.50 kV (ESI^−^); capillary temperature, 325 °C; MS1 range, *m*/*z* 100–1000; MS1 resolving power, 60,000 FWHM; number of data dependant scans per cycle, 4; MS/MS resolving power, 15,000 FWHM; normalized collision energy, 30%; dynamic exclusion time, automatic [36].

Raw mass spectrometry data were converted to mzXML format using MSConvert tool embedded in the Proteowizard software package (v3.0.8789). Peak detection, filtering and alignment were performed via the R package XCMS (v3.12.0) using the centWave algorithm. The main parameters were as follows: bw = 2, ppm = 15, peakwidth = c(5, 30), mzwid = 0.015, mzdiff = 0.01. The output was a metabolite intensity matrix. To correct for systematic variation, support vector regression normalization based on pooled quality control (QC) samples was applied. Metabolites with a relative standard deviation (RSD) > 30% in QC samples were excluded prior to downstream analyses.

### 2.7. Statistical Analysis

Plant growth traits and mineral nutrient contents were analyzed using one-way analysis of variance (ANOVA), followed by Duncan’s multiple range test to compare differences among treatments (*p* < 0.05). All statistical analyses were performed in SPSS 26.0 (IBM Corp., Armonk, NY, USA).

Multivariate statistical analyses were performed using the R ropls (v1.22.0) package. Principal component analysis (PCA) and orthogonal partial least squares discriminant analysis (OPLS-DA) were implemented for dimensionality reduction. Permutation tests were conducted to assess overfitting of the OPLS-DA models. R^2^X and R^2^Y represent the explanatory capacity of the constructed model for the X and Y matrices, respectively, whereas Q^2^ indicates the predictive power of the model. Values closer to 1 suggest superior model fitness and more accurate classification of training set samples. The influence and explanatory capacity of each metabolite for sample discrimination were evaluated using *p* values, variable importance in projection (VIP) derived from OPLS-DA, and fold change, facilitating the screening of candidate marker metabolites. Metabolites with *p* < 0.05 and VIP > 1 were considered significantly differentially accumulated metabolites.

Functional pathway enrichment and topological analysis of identified differential metabolites were carried out using MetaboAnalyst 6.0 [37]. Enriched metabolic pathways were identified through pathway topology analysis against the *Solanum lycopersicum* database. Key pathways were visualized by mapping differentially accumulated metabolites onto reference maps using the KEGG Mapper tool.

## 3. Results

### 3.1. Effect of GVL on Tomato Seedling Growth and Nutrient Accumulation

Application of GVL stimulated tomato seedling growth, with responses varying by concentration (Table 1). Compared to the control, the 0.25 g/L GVL treatment significantly increased plant height, stem diameter, shoot fresh weight, and shoot dry weight by 28.94%, 16.81%, 77.31%, and 100.51%, respectively. The 0.5 g/L GVL treatment also significantly enhanced these parameters, yielding increases of 20.19%, 13.53%, 58.28%, and 53.06%, respectively.

Root system development responded positively to GVL application, although the pattern of biomass allocation differed between treatments (Table 2). Compared with the control, both 0.25 g/L and 0.5 g/L GVL treatments significantly increased root length and the number of root tips. The 0.25 g/L GVL treatment exerted the strongest promotive effect on root biomass accumulation and surface area expansion, under which root surface area, root fresh weight, and root dry weight were all significantly higher than those of the control.

Shoot nutrient profiles varied with GVL dosage (Table 3). Application of 0.25 g/L GVL significantly enhanced shoot nitrogen (N) content by 18.64% compared to the control, whereas magnesium (Mg) contents decreased by 10.89%. The 0.5 g/L GVL treatment elicited a similar 17.16% increase in N contents but significantly reduced calcium (Ca) and Mg contents by 66.73% and 16.14%, respectively.

### 3.2. Multivariate Statistical Analysis of Metabolites in Tomato Seedling Leaves

Leaf metabolite profiles were analyzed by UHPLC-MS/MS after treatment with 0.25 and 0.5 g/L GVL at 24 h and 48 h post-application. Total ion chromatograms displayed well-defined metabolite peaks primarily within the 1–12 min retention time window (Figure S1). Quality control samples clustered tightly within the 95% confidence ellipse in the multivariate models (Figure S2, red dots), confirming high instrumental stability and good reproducibility across the analytical batch.

**Figure 1:**
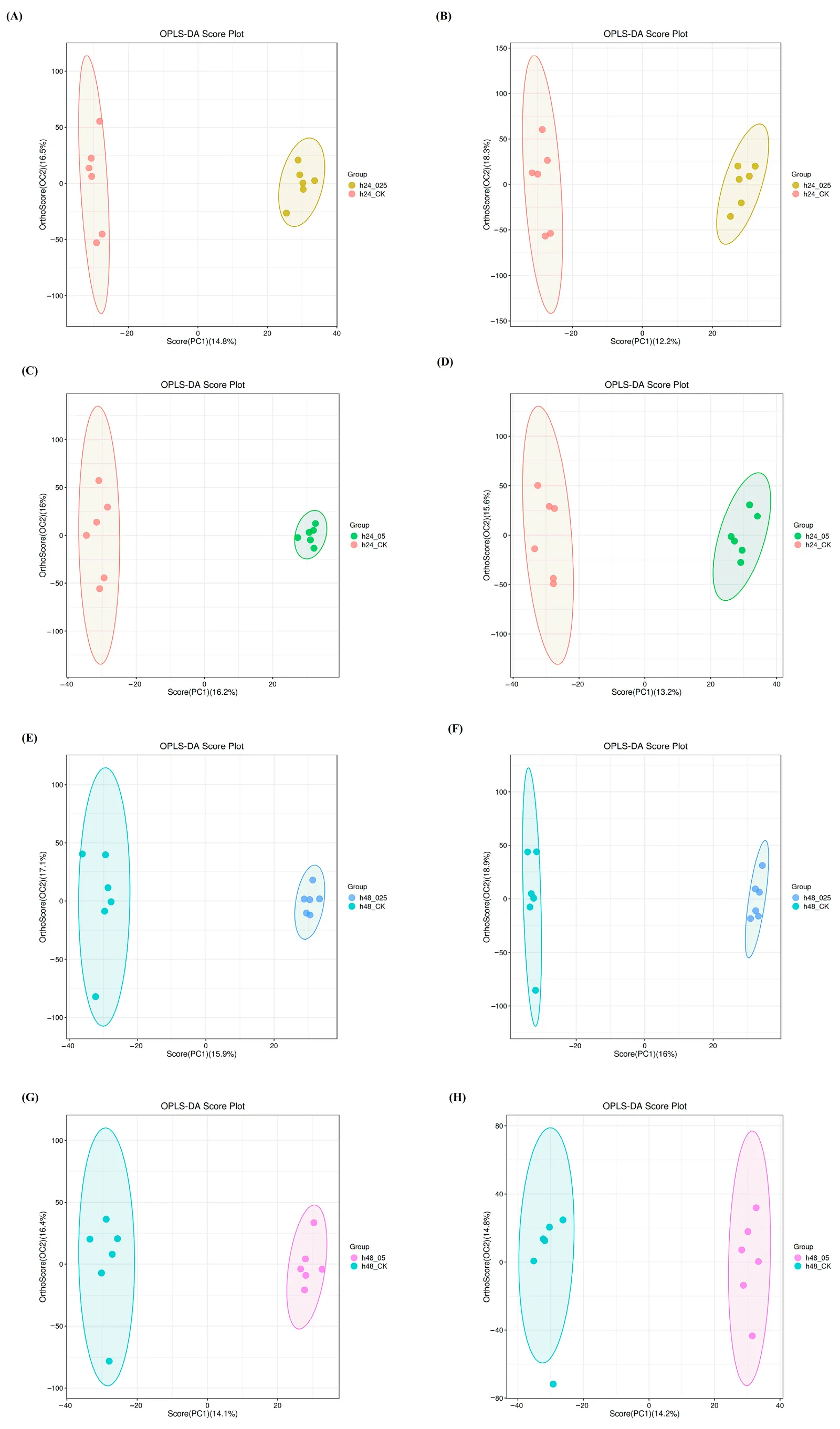
Orthogonal partial least squares-discriminant analysis score plots of metabolites in tomato seedling leaves under different γ-valerolactone (GVL) treatments. (**A**,**C**,**E**,**G**) Positive-ion mode score plots comparing the control (CK) group with GVL-treated groups at 0.25 g/L for 24 h, 0.5 g/L for 24 h, 0.25 g/L for 48 h, and 0.5 g/L for 48 h, respectively. (**B**,**D**,**F**,**H**) Corresponding score plots acquired in negative-ion mode. Each point represents a biological replicate, and ellipses denote the 95% confidence region.

Integrated multivariate analysis, incorporating PCA (Figure S3) and OPLS-DA (Figure 1), revealed that exogenous GVL application markedly remodeled the foliar metabolome. In the PCA score plot, control (CK) samples exhibited tight clustering under all time points, reflecting high intragroup reproducibility. While most treatment samples grouped closely, slight dispersion was observed in a few individuals, likely stemming from inherent biological variability or minor technical variation. Notably, samples from GVL-treated groups formed discrete clusters that were clearly separated from the CK group, indicating substantial treatment-driven metabolic shifts.

To maximize class separation, supervised OPLS-DA models were constructed (Table 4, Figure 1). Model performance metrics confirmed robustness, with R^2^Y and Q^2^ values consistently exceeding 0.5. The models achieved complete segregation of treatment and control groups into non-overlapping clusters, reinforcing the pronounced effect of GVL on leaf metabolism. Interestingly, Q^2^ value tended to increase with treatment duration (24 h vs. 48 h), suggesting that prolonged GVL exposure intensified metabolic divergence. Collectively, the combined PCA and OPLS-DA analyses effectively delineated metabolic distinctions among the four experimental groups, supporting the validity of subsequent differential metabolite screening.

### 3.3. Metabolite Profiling and Identification of Differentially Accumulated Metabolites

**Figure 2:**
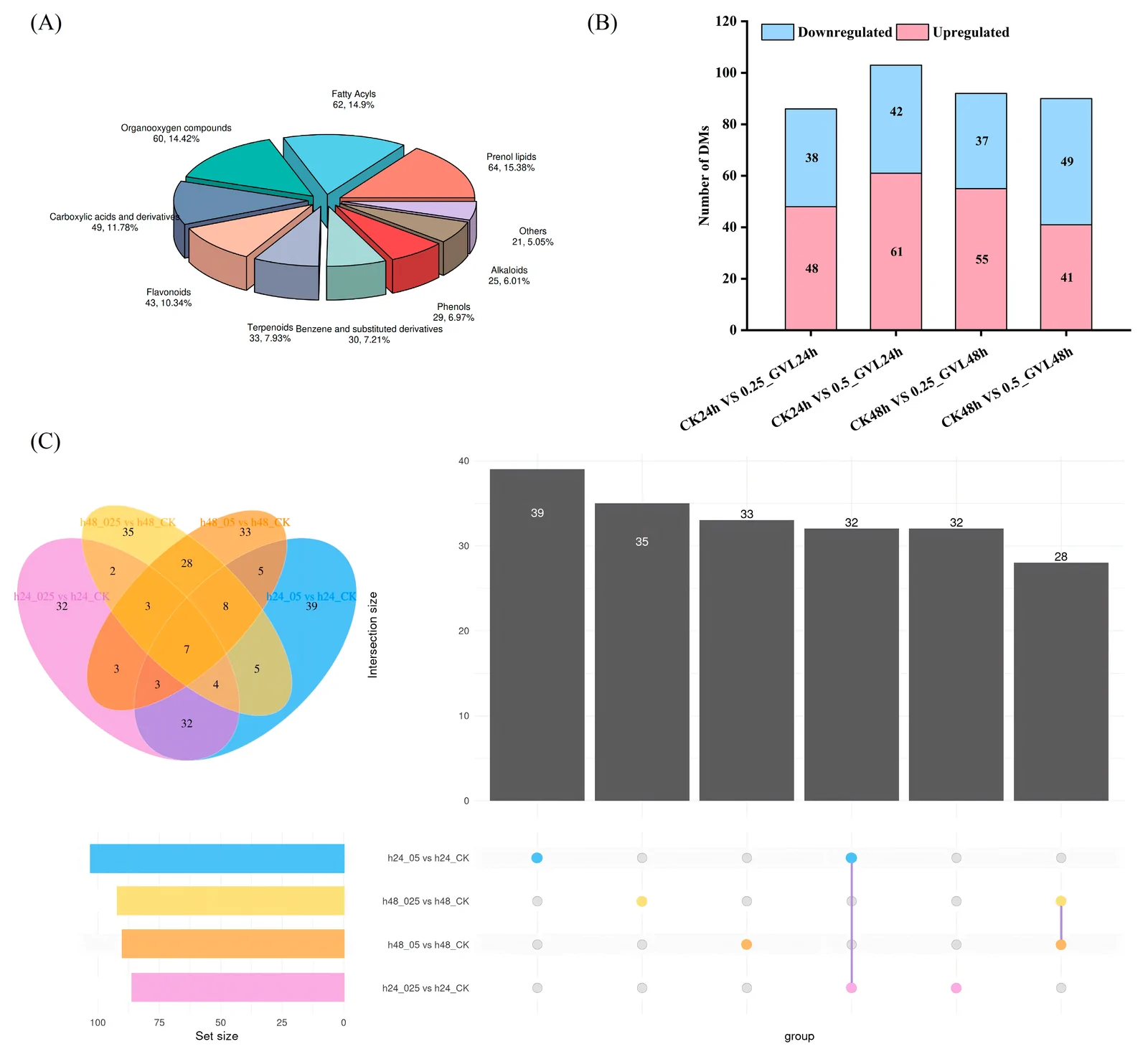
Metabolite profiling and differential accumulation in tomato seedling leaves following γ-valerolactone (GVL) treatment. (**A**) Classification of identified metabolites based on chemical ontology. (**B**) Bar plot depicting the number of differentially accumulated metabolites (DAMs) between the control (CK) and GVL-treated groups at 24 h and 48 h (0.25 g/L and 0.5 g/L). Upregulated and downregulated metabolites are indicated in pink and blue, respectively. (**C**) Overlap of DAMs between the control (CK) and GVL-treated groups at 24 h and 48 h (0.25 g/L and 0.5 g/L), as illustrated by a Venn diagram (**left**) and a bar chart indicating set sizes (**right**). h24_CK or h48_CK represents the non-GVL control at 24 or 48 h post treatment; h24_025 or h48_025 represents the 0.25 g/L GVL control at 24 or 48 h post treatment; h24_05 or h48_05 represents the 0.5 g/L GVL control at 24 or 48 h post treatment.

To characterize the metabolic reprogramming of tomato seedlings in response to GVL, a comprehensive profiling of leaf extracts was conducted using UHPLC-MS/MS. A total of 703 distinct metabolites were identified and annotated across all experimental groups (Figure 2A). Based on chemical ontology, these metabolites were categorized into several major classes. Prenol lipids (15.38%), fatty acyls (14.9%), and organooxygen compounds (14.42%) represented the dominant fractions, followed by carboxylic acids and derivatives (11.78%) and flavonoids (10.34%). This compositional overview establishes a metabolic baseline that defines the foliar biochemical landscape under GVL exposure.

Differentially accumulated metabolites (DAMs) identified distinct metabolic responses contingent on GVL concentration and exposure time. At 24 h post-treatment, the 0.25 g/L GVL application upregulated 48 metabolites and downregulated 38 relative to the control. The 0.5 g/L treatment at the same time point elicited more extensive changes, with 61 metabolites upregulated and 42 downregulated. By 48 h, the 0.25 g/L treatment resulted in 55 upregulated and 37 downregulated metabolites, whereas the 0.5 g/L treatment shifted the balance, yielding 41 upregulated and 49 downregulated metabolites (Figure 2B). The most pronounced response occurred at 24 h under 0.5 g/L GVL, with a total of 103 significantly altered metabolites. Substantial metabolic shifts were also evident following 48 h of 0.25 g/L treatment (91 DAMs). Notably, even the lowest exposure level (0.25 g/L at 24 h) triggered alterations in 86 metabolites, underscoring the sensitivity of tomato seedlings to GVL.

Venn diagram and UpSet plot analyses further clarified these dynamics. The 24 h 0.5 g/L treatment group harbored the largest unique set of DAMs. Notably, seven metabolites were consistently dysregulated across all four treatment groups, suggesting a core metabolic signature of GVL exposure. A high degree of overlap was also observed between the 24 h and 48 h sampling points within the 0.25 g/L treatment, indicating sustained modulation of specific pathways. These results demonstrate that GVL-induced metabolic reprogramming is strongly time- and dose-dependent (Figure 2C).

**Figure 3:**
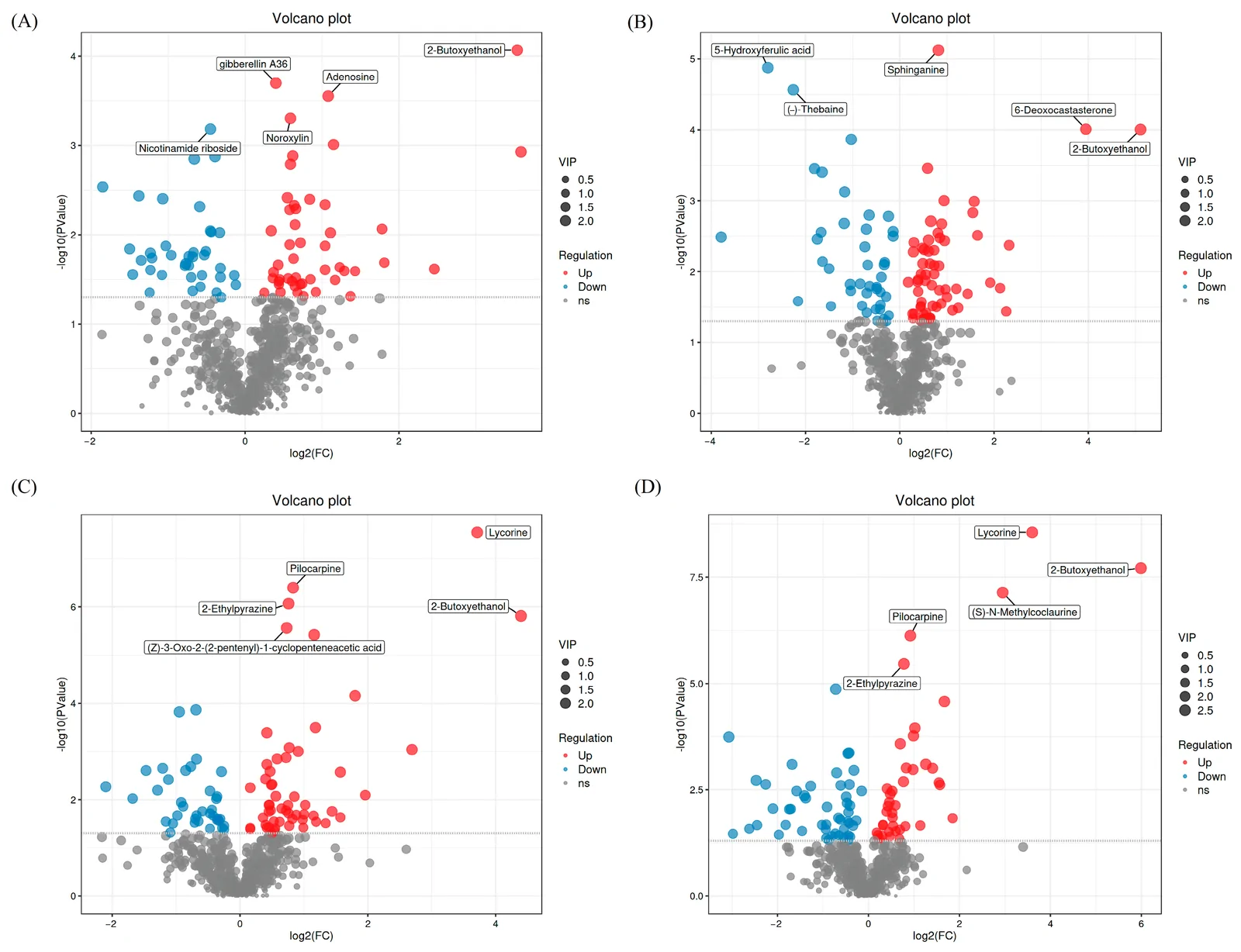
Volcano plots showing differentially accumulated metabolites (DAMs) in tomato seedling leaves under different concentrations of γ-valerolactone (GVL) treatment at different time points, comparing GVL-treated and non-treated (CK) samples. (**A**) CK vs. 0.25 g/L GVL treatment for 24 h. (**B**) CK vs. 0.5 g/L GVL treatment for 24 h. (**C**) CK vs. 0.25 g/L GVL treatment for 48 h. (**D**) CK vs. 0.5 g/L GVL treatment for 48 h. Red dots denote significantly up regulated metabolites, and blue dots denote down regulated metabolites. The horizontal dashed line indicates the significance threshold (*p* < 0.05), and metabolites with VIP ≥ 1 and *p* < 0.05 were identified as DAMs.

**Figure 4:**
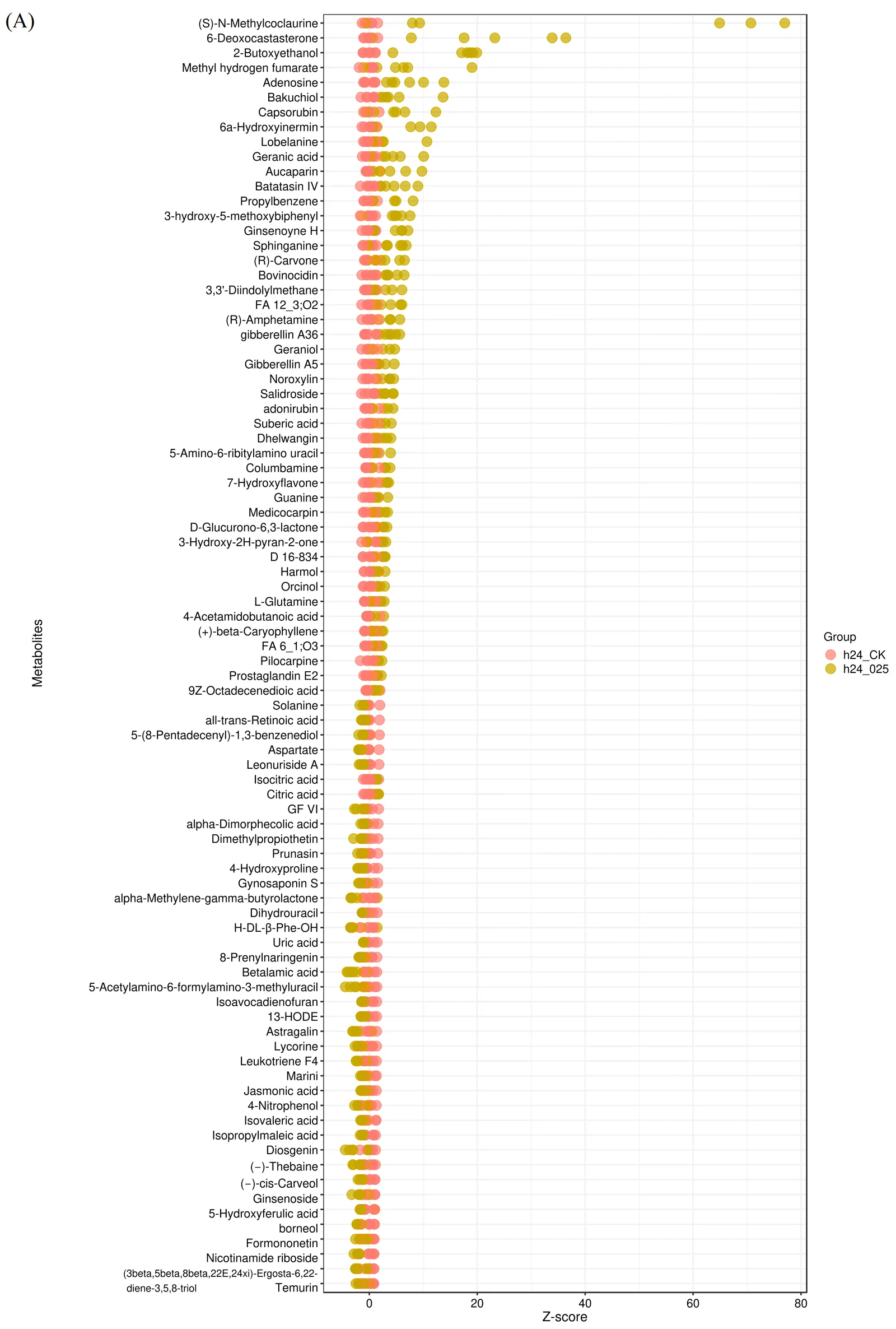

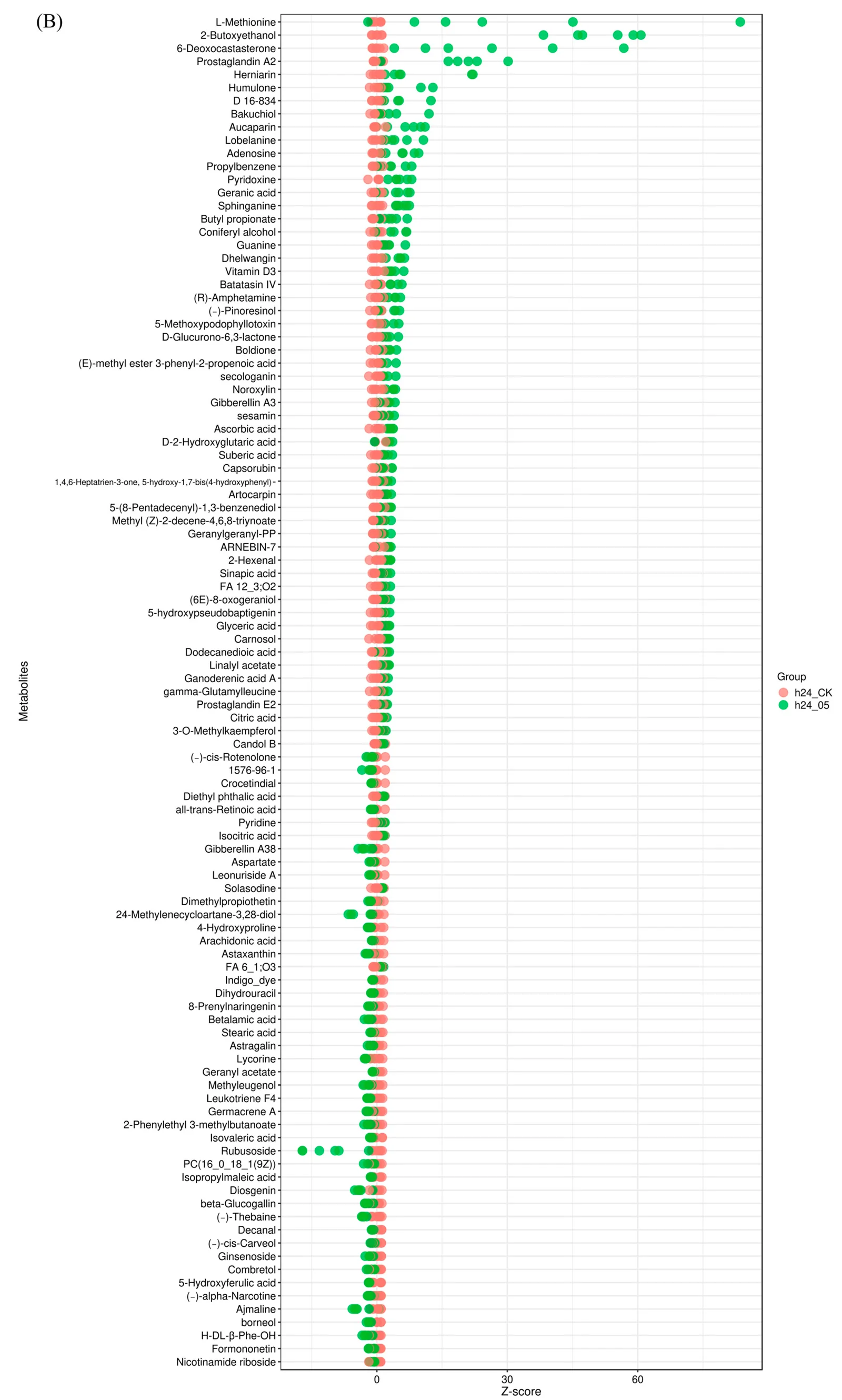

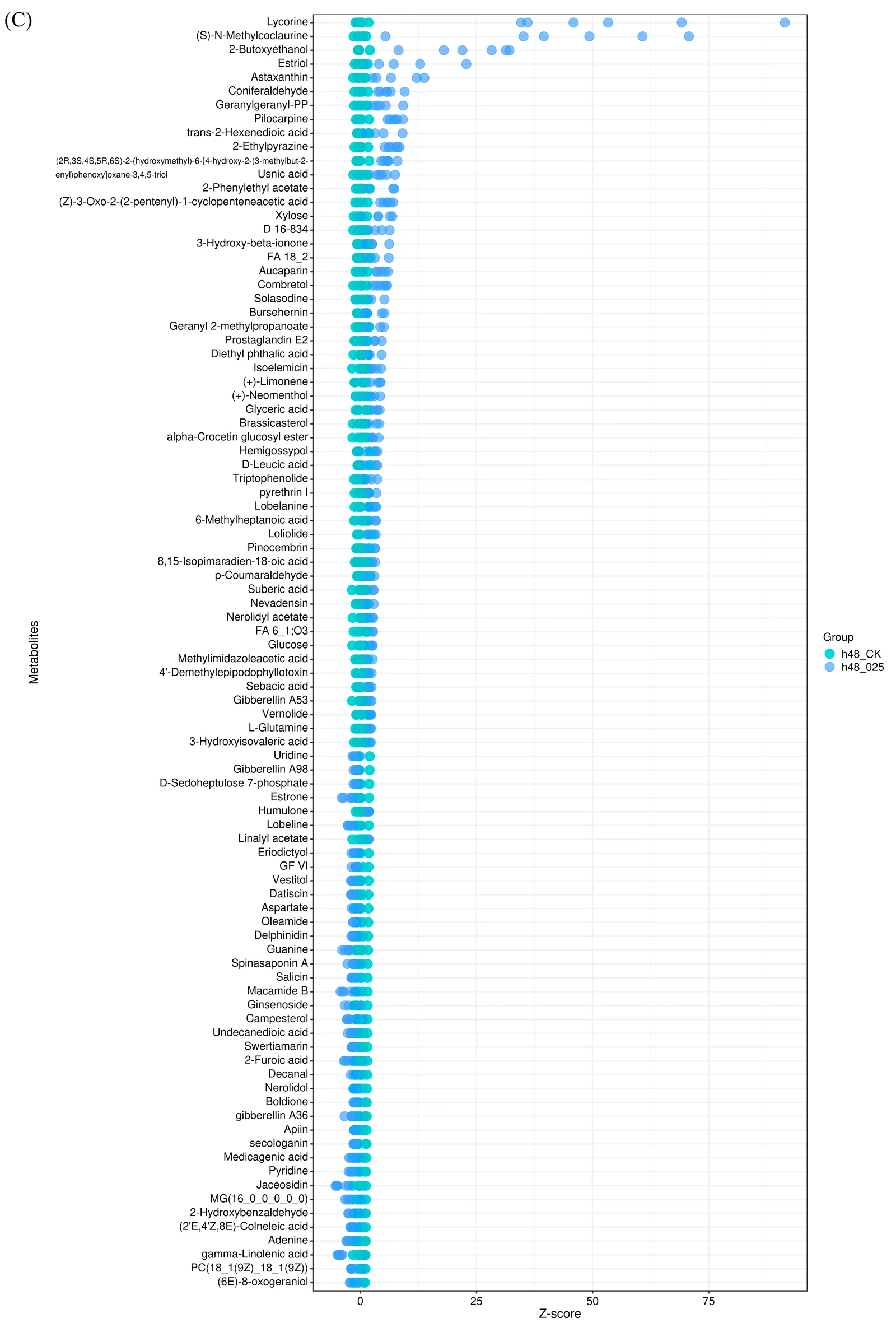

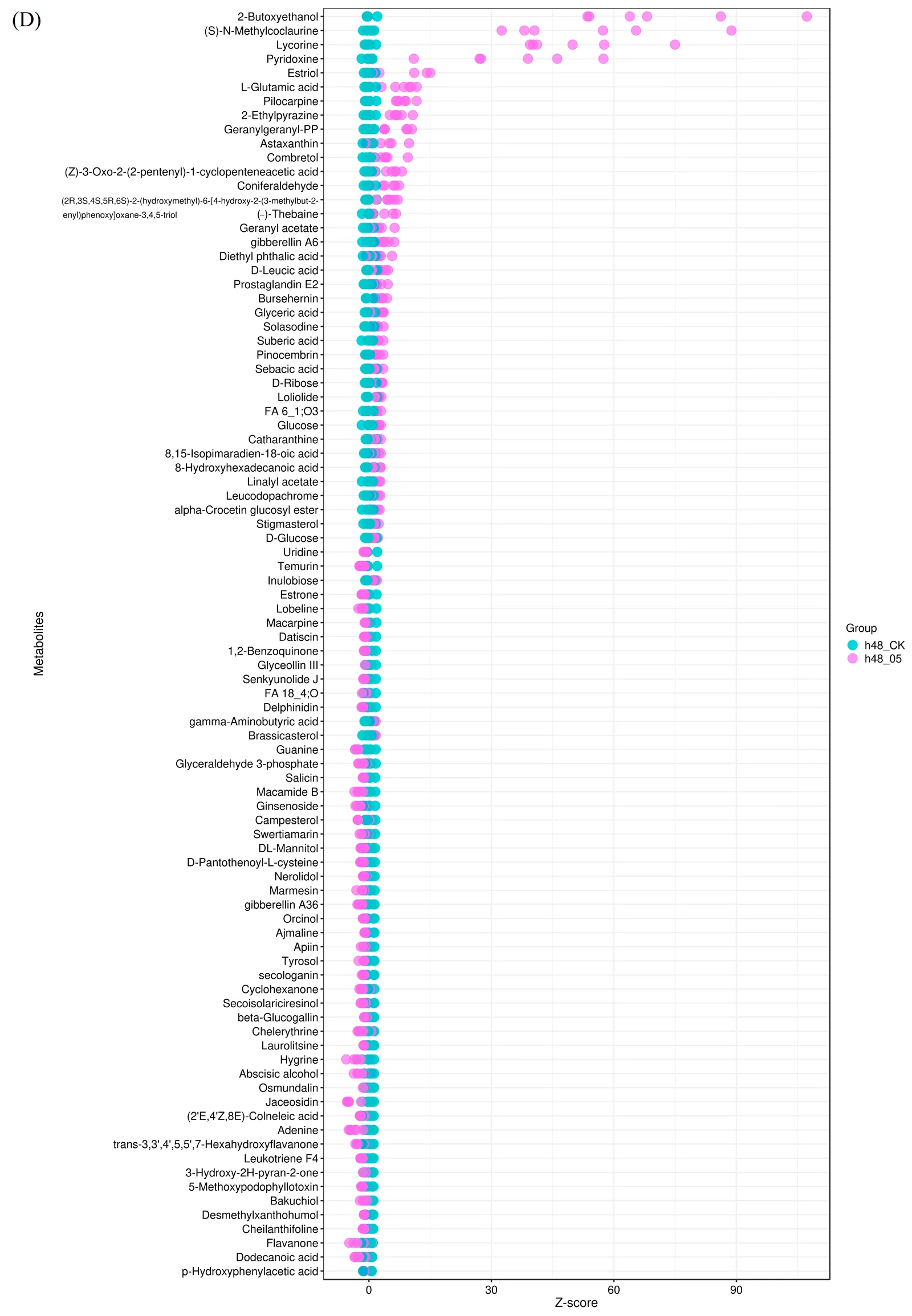
Z-score plots illustrating the relative abundance of differentially accumulated metabolites (DAMs) in tomato seedling leaves under different concentrations of γ-valerolactone (GVL) treatment at various time points, comparing GVL-treated and non-treated (CK) samples. (**A**) CK vs. 0.25 g/L GVL for 24 h. (**B**) CK vs. 0.5 g/L GVL for 24 h. (**C**) CK vs. 0.25 g/L GVL for 48 h. (**D**) CK vs. 0.5 g/L GVL for 48 h. Color intensity reflects the Z-score, representing the standard deviation of metabolite abundance relative to the mean across all samples.

To elucidate concentration- and time-dependent metabolic responses, we performed a comparative analysis of DAMs across all treatment groups. At 24 h post-treatment, 0.25 g/L GVL induced a moderate yet distinct metabolomic shift, characterized by a predominance of upregulated metabolites (Figure 3A). Key upregulated compounds included 2-butoxyethanol, adenosine, and gibberellin A36, although the latter two exhibited log2FC values proximal to the significance threshold. Nicotinamide riboside was the most significantly downregulated metabolite. Corresponding Z-score plots corroborated this trend, showing a rightward shift for most metabolites (e.g., 2-butoxyethanol, adenosine) in the treated group, whereas nicotinamide riboside displayed a marked leftward shift, indicating depletion (Figure 4A). This pattern suggests that even brief, low-dose GVL exposure initiates a predominantly stimulatory metabolic response in tomato leaves.

Increasing the concentration to 0.5 g/L GVL for 24 h elicited more extensive metabolic remodeling (Figure 3B). The volcano plot revealed a greater number of significant DAMs, particularly within the upregulated fraction. Sphinganine, 6-deoxocastasterone, and 2-butoxyethanol emerged as key upregulated metabolites, while 5-hydroxyferulic acid and (−)-thebaine were notably suppressed. Z-score analysis confirmed these trends, displaying a pronounced rightward shift for the majority of metabolites in the treated group, which underscores the robust inductive effect of higher GVL doses on leaf metabolism (Figure 4B).

Prolonged exposure (48 h) continued to drive metabolic reprogramming, albeit with distinct quantitative profiles. Under 0.25 g/L GVL, alkaloids such as pilocarpine and 2-ethylpyrazine were markedly elevated (Figure 3C and Figure 4C). The most extensive metabolic perturbation occurred with 0.5 g/L GVL at 48 h. The volcano plot displayed a dense cluster of significant DAMs, featuring exceptional upregulation of (S)-N-methylcoclaurine, pilocarpine, and 2-butoxyethanol, often exhibiting remarkably high fold changes (Figure 3D). Z-score plots highlighted the magnitude of this response, with a pronounced rightward shift across numerous metabolites, indicating that high-concentration, long-duration GVL exposure not only sustains but amplifies metabolic alterations over time (Figure 4D).

### 3.4. KEGG Enrichment Analysis of Differentially Accumulated Metabolites (DAMs)

To explore the metabolic networks underlying the growth-promoting effects of GVL, KEGG pathway enrichment analysis was performed on DAMs identified at 24 and 48 h post-treatment. A total of 70 distinct KEGG pathways were annotated across the four treatment comparisons (Table S1). Pathways with *p* < 0.05 were considered significantly enriched, while those with 0.05 ≤ *p* < 0.10 were regarded as showing enrichment trends.

**Figure 5:**
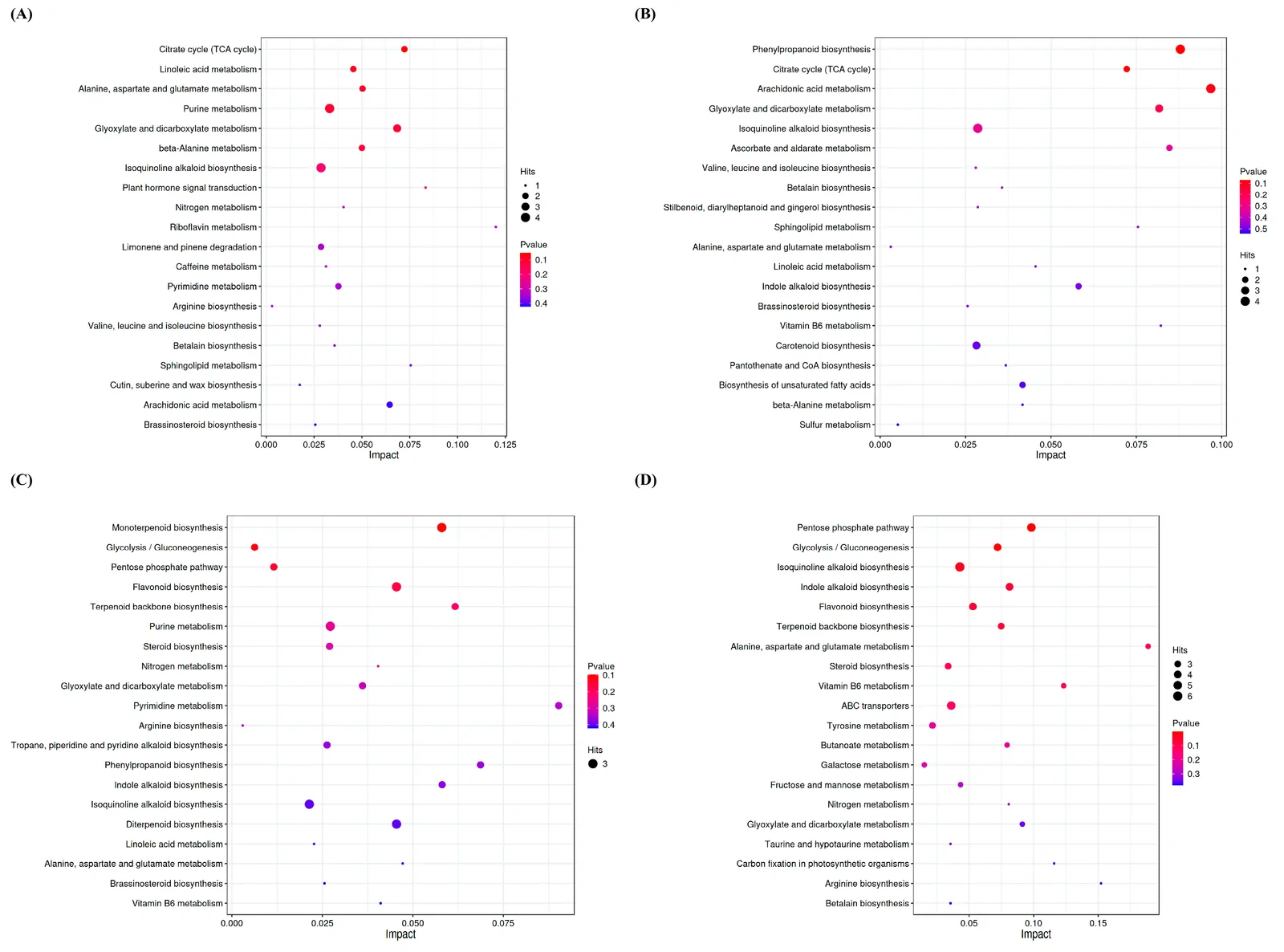
KEGG pathway enrichment analysis of differentially accumulated metabolites (DAMs) in tomato seedling leaves treated with γ-valerolactone (GVL) at different concentrations and sampling time points, comparing GVL-treated and non-treated (CK) samples. (**A**) CK vs. 0.25 g/L GVL for 24 h. (**B**) CK vs. 0.5 g/L GVL for 24 h. (**C**) CK vs. 0.25 g/L GVL for 48 h. (**D**) CK vs. 0.5 g/L GVL for 48 h. The horizontal axis represents the pathway impact score, and the vertical axis lists the enriched KEGG pathways. The size of each node is proportional to the number of DAMs mapped to the corresponding pathway, while the color gradient indicates the −log_10_(*p*-value), with red denoting higher statistical significance and blue denoting lower significance.

**Figure 6:**
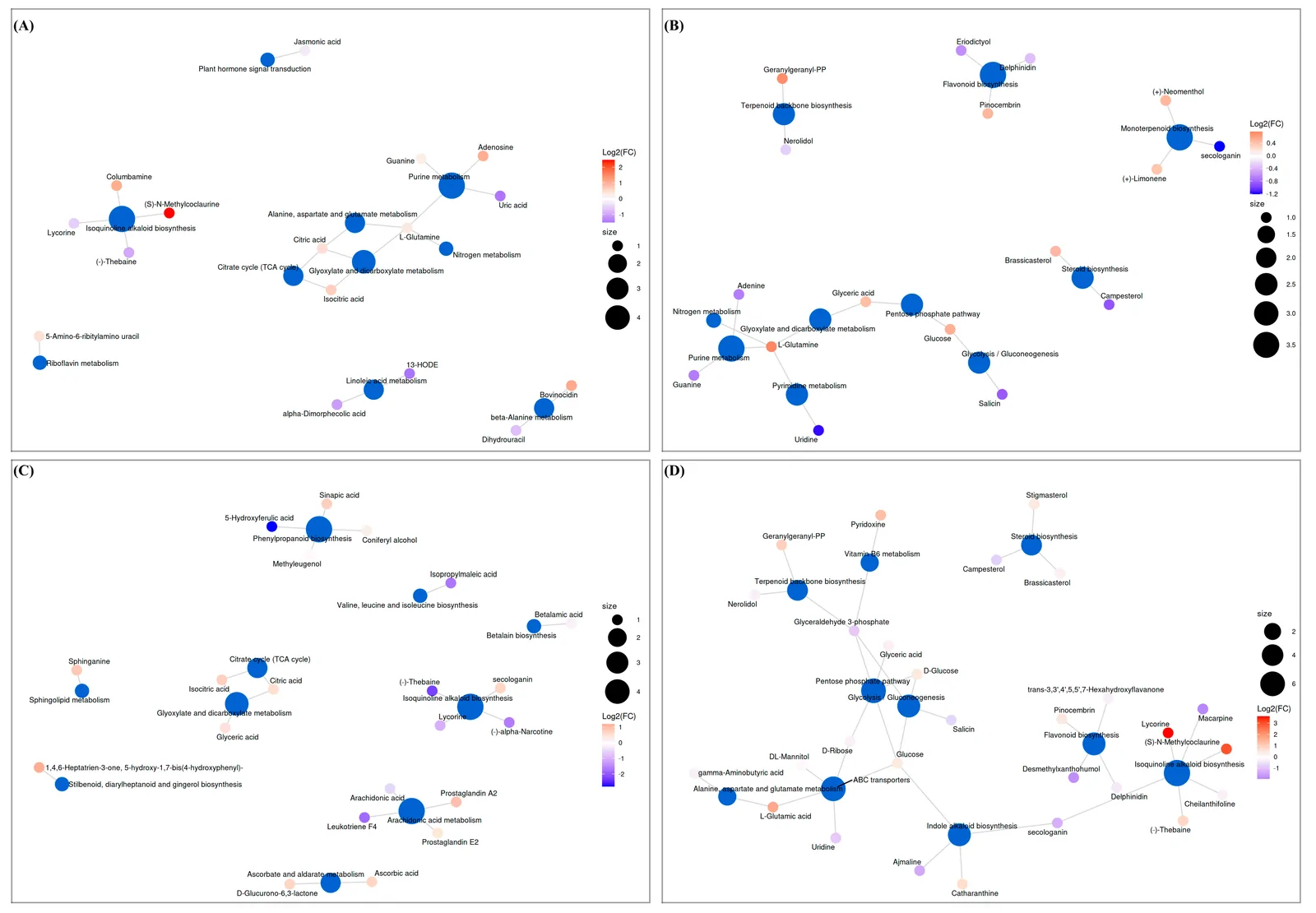
Visualization analysis of metabolite-pathway association networks for tomato seedling leaves treated with γ-valerolactone (GVL) at different concentrations and sampling time points, comparing GVL-treated and non-treated (CK) samples. (**A**) CK vs. 0.25 g/L GVL for 24 h. (**B**) CK vs. 0.5 g/L GVL for 24 h. (**C**) CK vs. 0.25 g/L GVL for 48 h. (**D**) CK vs. 0.5 g/L GVL for 48 h. Circular nodes represent KEGG pathways, and the node diameter is proportional to the degree of connectivity (i.e., the number of associated differentially accumulated metabolites). Metabolite coloration is based on the log_2_(fold change), with red indicating upregulation and blue indicating downregulation; darker shades correspond to larger magnitudes of change.

At 24 h, no significantly enriched pathways (*p* < 0.05) were identified in either treatment group. Nevertheless, several pathways exhibited enrichment trends (*p* < 0.10). In the 0.25 g/L GVL group, these included the tricarboxylic acid (TCA) cycle (*p* = 0.053), linoleic acid metabolism (*p* = 0.095), and alanine, aspartate and glutamate metabolism (*p* = 0.095) (Figure 5A, Figure 6A and Figure S4A). For the 0.5 g/L GVL group, phenylpropanoid biosynthesis (*p* = 0.072), the TCA cycle (*p* = 0.077), and arachidonic acid metabolism (*p* = 0.095) showed enrichment trends (Figure 5B, Figure 6B and Figure S4B).

Extending the exposure to 48 h markedly expanded the scope of metabolic reprogramming. In the comparison between CK and 0.25 g/L GVL, monoterpenoid biosynthesis showed a trend toward enrichment (*p* = 0.097), along with glycolysis/gluconeogenesis (*p* = 0.113) and the pentose phosphate pathway (PPP) (*p* = 0.138) (Figure 5C, Figure 6C and Figure S4C). Similarly, the 0.5 g/L GVL group at 48 h was the only condition where pathways reached statistical significance (*p* < 0.05), with four significantly enriched pathways: pentose phosphate pathway, glycolysis/gluconeogenesis, isoquinoline alkaloid biosynthesis, and indole alkaloid biosynthesis (Figure 6D and Figure S4D; Table 5). Notably, bubble plot analysis (Figure 5D) revealed that pathways enriched under the 0.5 g/L GVL treatment at 48 h exhibited lower *p*-values and higher impact values compared to those in the 0.25 g/L group. This suggests that higher concentrations of GVL induce more robust and targeted metabolic remodeling, particularly affecting central carbon metabolism and specialized secondary metabolite biosynthesis.

## 4. Discussion

### 4.1. GVL as a Novel Microbial-Derived Plant Biostimulant

GVL is a volatile organic compound derived from sustainable natural biomass that is readily biodegradable and exhibits minimal environmental [38] and human health impacts throughout its lifecycle [39]. It is extensively utilized as a fuel additive, green solvent, and in fine chemical synthesis [40]. Recent studies employing solid-phase microextraction coupled with gas chromatography–mass spectrometry (SPME-GC-MS) have identified GVL as a key bioactive VOC emitted by PGPR *Stutzerimonas stutzeri* NRCB010, demonstrating remarkable growth-promoting properties [15]. To further elucidate the mechanisms underlying these effects, the present study integrated physiological assessments with a comprehensive, time-resolved metabolomic analysis of tomato seedlings exposed to varying GVL concentrations.

Accumulating evidence indicates that lactones function as potent biostimulants to promote plant growth. For instance, exogenous application of synthetic N-(3-oxo-octanoyl)-L-homoserine lactone significantly enhances growth parameters and root ion accumulation in citrus seedlings [16]. Similarly, epibrassinolide significantly promotes the growth and development of tomato seedlings [41]. Building on this context, our findings demonstrate that GVL significantly improves key physiological indices (including plant height, stem diameter, shoot and root biomass, and root morphological traits) and nitrogen uptake in tomato seedlings under greenhouse conditions (Table 1, Table 2 and Table 3). These results align closely with recent hydroponic studies reporting the growth-promoting effects of GVL [16] and extend them by characterizing the metabolic networks involved.

### 4.2. Modulation of Phytohormones and Signaling Metabolites

We observed that GVL treatment significantly increased foliar levels of gibberellin A36, 6-deoxocastasterone, 2-ethylpyrazine and other metabolites (Figure 3 and Figure 4). Gibberellins are canonical phytohormones regulating plant growth and development [42]. Brassinosteroids (BRs) are essential steroidal signaling molecules governing organ morphogenesis and physiological processes. As a member of the C19 steroid family, 6-deoxocastasterone serves as a biosynthetic precursor for other brassinosteroids, stimulates plant growth and development by facilitating root elongation, boosting chlorophyll accumulation, and enhances tolerance to environmental stresses [43]. Furthermore, the elevation of 2-ethylpyrazine, typically associated with flavor volatile biosynthesis, may reflect broader shifts in nitrogen metabolism or secondary metabolic branching induced by GVL. Collectively, the modulation of these signaling metabolites suggests that GVL primes the plant for enhanced growth and environmental responsiveness, including drought, salinity and extreme temperature stress.

Mechanistically, KEGG enrichment analysis revealed that GVL induced distinct metabolic reprogramming patterns contingent on both concentration and exposure duration. At 24 h, the lower GVL concentration (0.25 g/L) primarily enriched primary metabolic pathways, including the tricarboxylic acid (TCA) cycle, amino acid metabolism, and purine metabolism (Figure 5 and Figure 6 and Figure S4). These pathways supply the ATP and biosynthetic precursors necessary to sustain the elevated energetic demands of rapid growth. In contrast, the higher concentration (0.5 g/L) at this early time point preferentially activated phenylpropanoid biosynthesis. This shift toward secondary metabolism is consistent with previous transcriptomic findings suggesting GVL reinforces cell wall architecture and stress tolerance via phenylpropanoid metabolism [16].

Extending the exposure to 48 h revealed a convergence toward core carbon metabolism. Both concentrations significantly enriched glycolysis/gluconeogenesis and the pentose phosphate pathway (PPP), alongside specialized metabolite pathways such as flavonoid and alkaloid biosynthesis (Table 5). Notably, the 0.5 g/L treatment at 48 h triggered the most robust response, uniquely activating isoquinoline and indole alkaloid biosynthesis. This specific induction of nitrogen-containing specialized metabolites aligns with reports that brassinosteroids can upregulate genes involved in alkaloid biosynthesis to bolster plant defense [44]. Thus, while lower doses and shorter durations of GVL appear to optimize primary metabolism for growth, higher doses and prolonged exposure initiate a defensive metabolic remodeling, potentially enhancing the plant’s resilience to environmental stressors.

### 4.3. Limitations and Future Perspectives

Despite the insights gained, this study has several limitations. First, all experiments were conducted under controlled greenhouse conditions. The effects of GVL require validation in field environments across diverse soil types and climatic regions. Second, growth measurements were restricted to the seedling stage. Long-term tracking from seedling to harvest (including plant and root traits, nutrient uptake, etc.) is required. The effects of GVL on photosynthetic and antioxidant enzyme activities under abiotic stresses also remain to be elucidated. Third, metabolomics only provides associations between GVL-induced metabolic changes and growth stimulation. Functional validation and causal evidence linking metabolic reprogramming to growth promotion are lacking, and upstream regulatory mechanisms remain unclear. Further research should perform functional perturbation of key metabolic pathways and explore the crosstalk between GVL and endogenous phytohormone signaling (e.g., GA, BR, and JA/SA). Fourth, agronomic applicability and regulatory compliance require further evaluation before commercialization. Production costs, large-scale supply, compatibility with field practices, and systematic residue, food safety, and ecological risk assessments are necessary for registration as a biostimulant. Finally, future studies should investigate the synergistic potential of co-applying exogenous GVL with its producing strain *S. stutzeri* NRCB010, or combining GVL with other well-characterized PGPR such as *Pseudomonas* spp. and *Bacillus* spp., to develop composite biostimulant formulations for sustainable production of tomatoes and other high-value horticultural crops.

## 5. Conclusions

In this study, a widely targeted metabolomics approach based on ultra-high-performance liquid chromatography–tandem mass spectrometry (UHPLC-MS/MS) was employed to systematically characterize the metabolic reprogramming of tomato seedling leaves following exposure to γ-valerolactone (GVL) at two concentrations (0.25 and 0.5 g/L) over two time intervals (24 and 48 h). Physiological assessments confirmed that GVL significantly promoted both shoot and root growth. The 0.25 g/L treatment exerted the most pronounced stimulatory effects, increasing shoot height, fresh weight, and dry weight by 28.94%, 77.31%, and 100.51%, respectively, relative to the untreated control, while also enhancing nitrogen accumulation in plant tissues. Exogenous GVL induced substantial, concentration- and time-dependent remodeling of the foliar metabolome. Differential metabolite screening revealed that low-concentration GVL (0.25 g/L) primarily triggered the accumulation of growth-promoting phytohormones, including gibberellin A36 and 6-deoxocastasterone. Conversely, high-concentration GVL (0.5 g/L) preferentially upregulated defense-associated alkaloids, such as (S)-N-methylcoclaurine and pilocarpine. KEGG pathway analysis further elucidated these divergent regulatory patterns. At 24 h, GVL primarily enriched primary metabolic pathways, specifically the TCA cycle, amino acid metabolism, and purine metabolism, providing the carbon skeletons and ATP necessary to support rapid growth. By 48 h, central carbon metabolism, including glycolysis/gluconeogenesis and the pentose phosphate pathway, was robustly activated alongside the biosynthesis of terpenoids and flavonoids. Notably, 48 h of treatment with 0.5 g/L GVL uniquely activated the biosynthesis of isoquinoline and indole alkaloids, indicating that high-dose GVL orchestrates a defensive metabolic shift to enhance environmental adaptability. Collectively, these findings demonstrate that GVL, a biodegradable compound derived from lignocellulosic biomass, synergistically modulates primary and secondary metabolic networks in a dose- and time-dependent manner, concurrently boosting growth and stress tolerance. This work provides robust metabolomic evidence supporting the development of GVL as a novel, bio-based plant biostimulant. Future studies should prioritize multi-environment field trials to validate agronomic efficacy and dissect the interplay between GVL, endogenous hormone signaling, nutrient uptake and rhizosphere microbiota.

## Figures and Tables

**Table 1:** Effects of γ-valerolactone (GVL) on shoot growth parameters of tomato seedlings.

GVL Concentration (g/L)	Plant Height (cm)	Stem Diameter (mm)	Shoot Fresh Weight (g)	Shoot Dry Weight (mg)
0	4.67 ± 0.21 b	3.20 ± 0.17 b	1.91 ± 0.06 b	308 ± 12 c
0.25	6.03 ± 0.23 a	3.74 ± 0.11 a	3.38 ± 0.16 a	617 ± 31 a
0.5	5.62 ± 0.13 a	3.64 ± 0.11 a	3.02 ± 0.16 a	471 ± 25 b

Note: Values are presented as means ± standard error, *n* = 6. Different letters after the values of the same column mean significant differences between treatments by Duncan’s post hoc tests (at *p* < 0.05).

**Table 2:** Effects of γ-valerolactone (GVL) on root morphological traits of tomato seedlings.

GVL Concentration (g/L)	Root Length (cm)	Root Surface Area (cm^2^)	Number of Root Tips	Root Fresh Weight (mg)	Root Dry Weight (mg)
0	38.8 ± 2.9 b	5.82 ± 0.49 b	225 ± 18 b	300 ± 29 b	32.4 ± 3.7 b
0.25	61.0 ± 7.3 a	8.43 ± 0.82 a	351 ± 34 a	372 ± 57 a	54.4 ± 5.6 a
0.5	62.1 ± 7.3 a	8.13 ± 1.04 ab	407 ± 42 a	351 ± 28 ab	41.7 ± 3.4 ab

Note: Values are presented as means ± standard error, *n* = 6. Different letters after the values of the same column mean significant differences between treatments by Duncan’s post hoc tests (at *p* < 0.05).

**Table 3:** Influence of γ-valerolactone (GVL) application on mineral nutrient contents in tomato shoots.

GVL Concentration (g/L)	Nitrogen (g/kg)	Phosphorus (g/kg)	Potassium (g/kg)	Calcium (g/kg)	Magnesium (g/kg)
0	19.58 ± 0.48 b	2.92 ± 0.11 a	12.85 ± 1.04 a	16.87 ± 0.77 a	5.18 ± 0.25 a
0.25	23.23 ± 0.59 a	2.63 ± 0.14 a	10.25 ± 1.04 a	15.12 ± 0.63 ab	4.62 ± 0.14 b
0.5	22.94 ± 0.54 a	2.69 ± 0.04 a	10.21 ± 0.58 a	14.93 ± 0.26 b	4.34 ± 0.12 b

Note: Values are presented as means ± standard error, *n* = 6. Different letters after the values of the same column mean significant differences between treatments by Duncan’s post hoc tests (at *p* < 0.05).

**Table 4:** Parameters of the orthogonal partial least squares-discriminant analysis models for tomato seedling leaves under different γ-valerolactone (GVL) treatments.

Comparison	Positive Mode			Negative Mode		
	R^2^X (cum)	R^2^Y (cum)	Q^2^ (cum)	R^2^X (cum)	R^2^Y (cum)	Q^2^ (cum)
h24_025 vs. h24_CK	0.313	0.996	0.574	0.304	0.995	0.486
h24_05 vs. h24_CK	0.321	0.996	0.649	0.289	0.993	0.563
h48_025 vs. h48_CK	0.33	0.995	0.675	0.452	0.999	0.715
h48_05 vs. h48_CK	0.305	0.994	0.647	0.29	0.994	0.685

Note: h24_CK or h48_CK represents the non-GVL control at 24 or 48 h post treatment; h24_025 or h48_025 represents the 0.25 g/L GVL control at 24 or 48 h post treatment; h24_05 or h48_05 represents the 0.5 g/L GVL control at 24 or 48 h post treatment. R^2^X represents the cumulative explanatory power of the model for the X dataset, R^2^Y denotes the cumulative explanatory power for the Y dataset, and Q^2^ reflects the predictive performance of the model. Values of R^2^ and Q^2^ closer to 1 indicate more stable and reliable models.

**Table 5:** Significantly enriched KEGG pathways (*p* < 0.05) of differentially accumulated metabolites (DAMs) in tomato seedling leaves under γ-valerolactone (GVL) treatments. 48h-05: 0.5 g/L GVL for 48 h.

Treatment	Pathway ID	Pathway Name	Total	Hits	*p* Value	Compound Name
48h-05	sly00030	Pentose phosphate pathway	35	5	0.001	Glucose; Glyceraldehyde 3-phosphate; D-ribose; D-Glucose; Glyceric acid
48h-05	sly00010	Glycolysis/Gluconeogenesis	31	4	0.003	Glucose; Glyceraldehyde 3-phosphate; D-Glucose; Salicin
48h-05	sly00950	Isoquinoline alkaloid biosynthesis	122	6	0.032	Secologanin; Cheilanthifoline; (S)-N-Methylcoclaurine; Macarpine; (−)-Thebaine; Lycorine
48h-05	sly00901	Indole alkaloid biosynthesis	70	4	0.048	Glucose; Secologanin; Ajmaline; Catharanthine

## Data Availability

The original contributions presented in this study are included in the article material. Further inquiries can be directed to the corresponding authors.

## Supplementary Materials

The following supporting information can be downloaded at: https://www.mdpi.com/article/10.3390/metabo16090621/s1, Table S1: KEGG-enriched metabolic pathways; Figure S1: Total ion chromatograms of tomato seedling leaves in positive and negative ion modes across different treatment groups; Figure S2: Principal component analysis (PCA) score plots of quality control (QC) and experimental samples; Figure S3: Principal component analysis score plots of tomato seedling leaf metabolites under γ-valerolactone (GVL) treatments; Figure S4: Bar plots illustrating KEGG pathway enrichment analysis of differentially accumulated metabolites in tomato seedling leaves treated with γ-valerolactone (GVL) at different concentrations and sampling time points, comparing GVL and non-GVL (CK) treated samples.

## Abbreviations

The following abbreviations are used in this manuscript:

GVL	γ-valerolactone
KAR1	Karrikin 1
PGPR	plant growth-promoting rhizobacterium
KEGG	Kyoto Encyclopedia of Genes and Genomes
UHPLC-MS/MS	ultra-high-performance liquid chromatography–tandem mass spectrometry
DAMs	differentially accumulated metabolites
