# Supplementary material for: γ-Valerolactone Reprograms Tomato Metabolism to Enhance Seedling Growth
Source: Metabolites. 2026 Aug 27;16(9):621. doi: 10.3390/metabo16090621 (PMC13608945; doi:10.3390/metabo16090621)
Supplement: Supplementary file 1 [file metabolites-16-00621-s001.zip › Supplementary Figures.pdf]

Supplementary figures

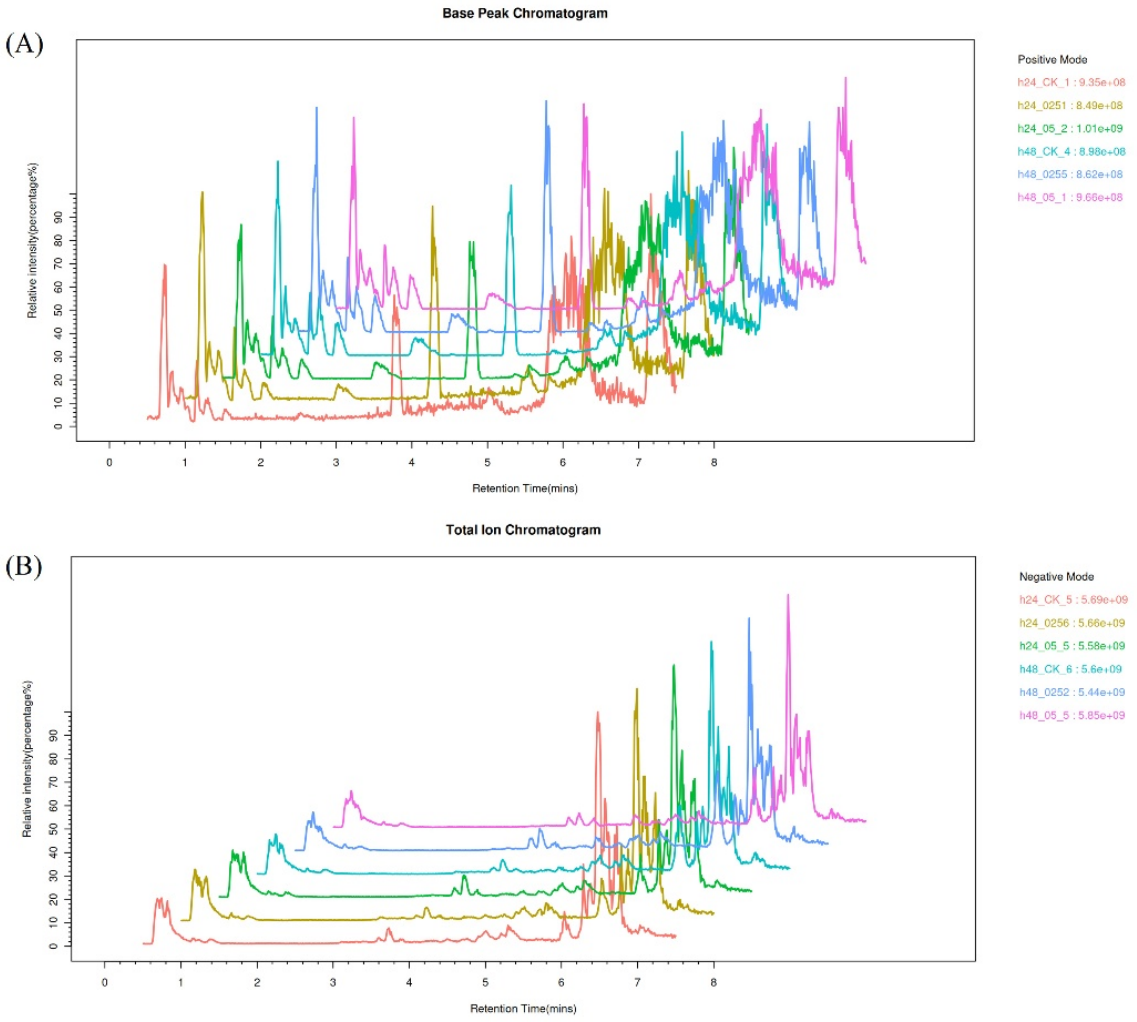

Figure S1. Total ion chromatograms (TICs) of tomato seedling leaves in positive and negative ion modes across different treatment groups. The *x-axis* represents the retention time (min), and the *y-axis* represents the ion intensity. The numerical value in the upper right corner of each panel indicates the maximum ion intensity for that sample. Different colors correspond to distinct treatment groups. (A) Positive ion mode; (B) Negative ion mode.

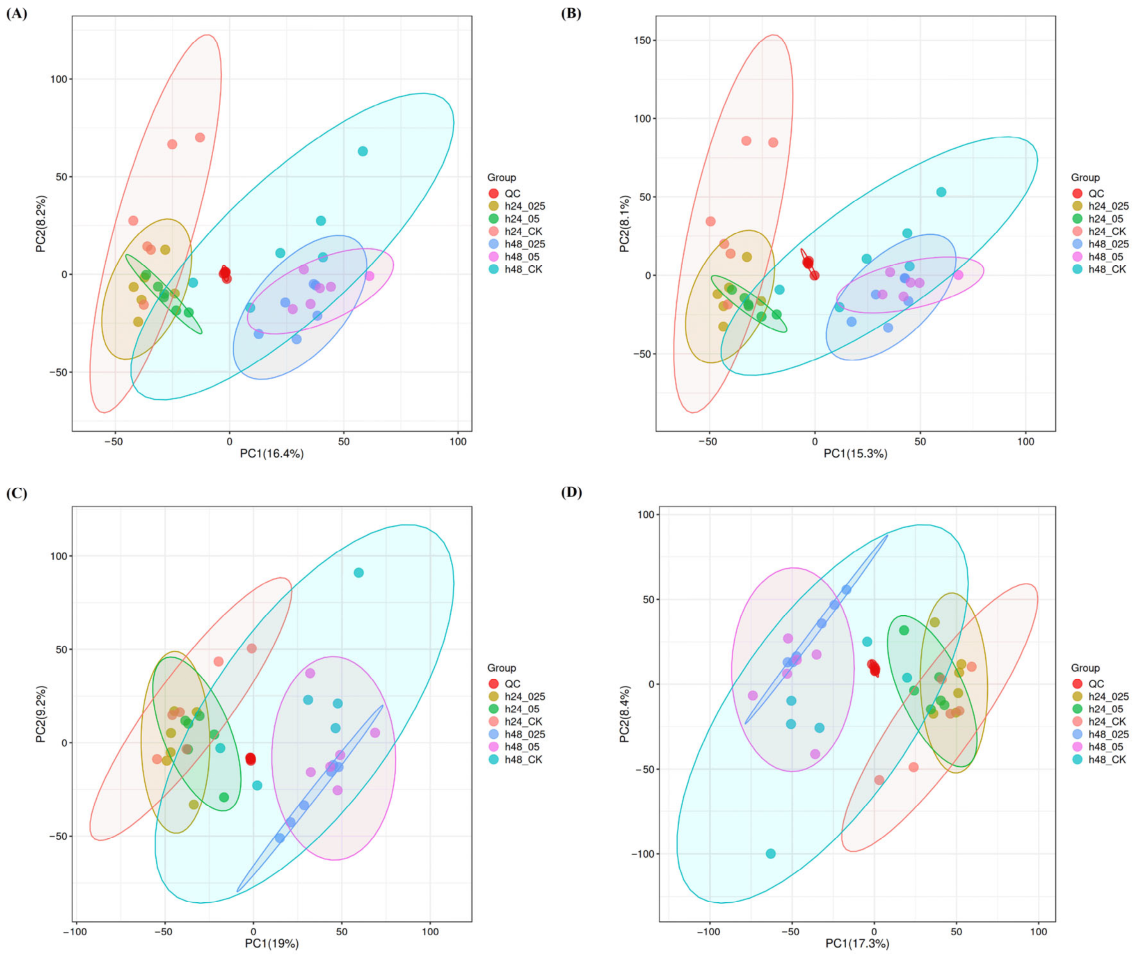

Figure S2. Principal component analysis (PCA) score plots of quality control (QC) and experimental samples. (A, C) PCA score plots of QC samples in positive and negative ion modes, respectively. (B, D) PCA score plots of all samples, including both experimental and QC samples, in positive and negative ion modes, respectively. QC samples are denoted by red dots, while colored dots represent experimental treatments. The tight clustering of QC samples within the 95% confidence ellipses indicates high instrumental stability and good reproducibility across the analytical batch.

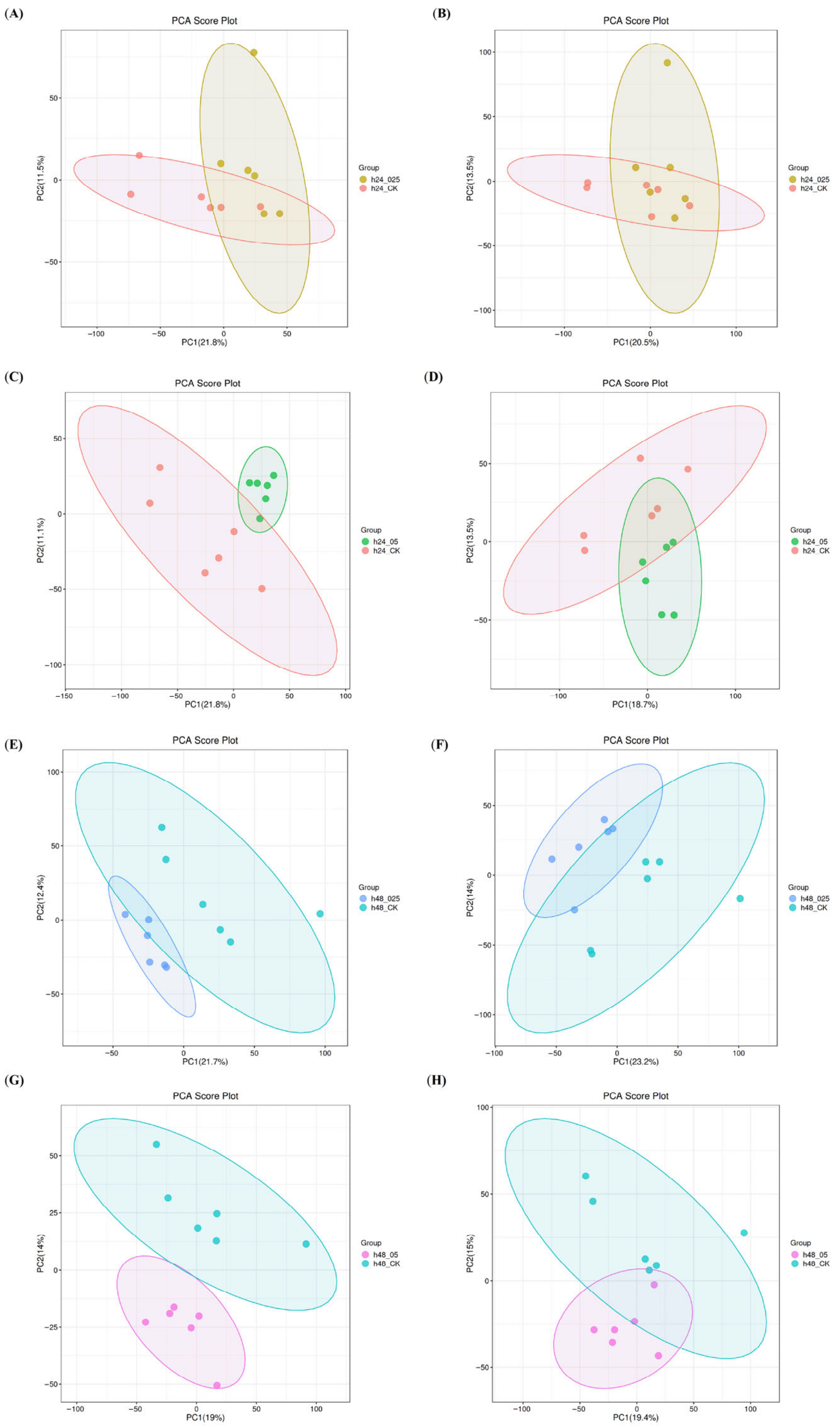

Figure S3. Principal component analysis (PCA) score plots of tomato seedling leaf metabolites under γ-valerolactone (GVL) treatments. The *x-axis* and *y-axis* represent the scores of the first (PC1) and second (PC2) principal components, respectively. Each point denotes an individual biological replicate, and ellipses indicate the 95% confidence region for each group. Different colors correspond to distinct treatment groups. (A, C, E, G) Positive ion mode comparisons: CK vs. 0.25 g/L GVL (24 h), CK vs. 0.5 g/L GVL (24 h), CK vs. 0.25 g/L GVL (48 h), and CK vs. 0.5 g/L GVL (48 h), respectively. (B, D, F, H) Corresponding comparisons acquired in negative ion mode.

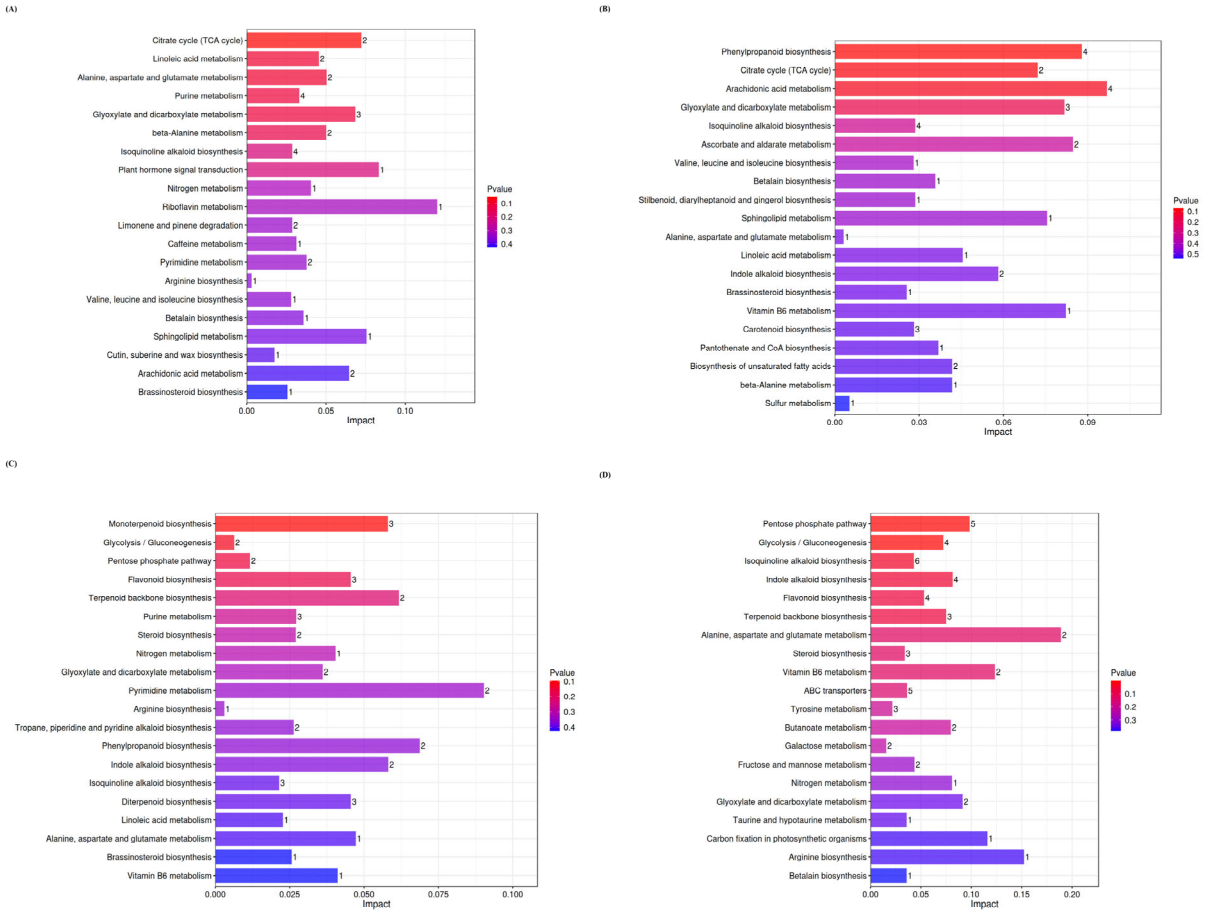

Figure S4. Bar plots illustrating KEGG pathway enrichment analysis of differentially accumulated metabolites (DAMs) in tomato seedling leaves treated with γ-valerolactone (GVL) at different concentrations and sampling time points, comparing GVL-treated and non-treated (CK) samples. (A) CK vs. 0.25 g/L GVL for 24 h. (B) CK vs. 0.5 g/L GVL for 24 h. (C) CK vs. 0.25 g/L GVL for 48 h. (D) CK vs. 0.5 g/L GVL for 48 h. The x-axis represents the pathway impact score derived from topological analysis, and the y-axis lists the significantly enriched KEGG pathways. The color gradient of the bars indicates the $-\log_{10}$ (p-value), with red denoting higher statistical significance and blue denoting lower significance. Numerical labels on the right side of each bar indicate the count of DAMs mapped to the respective pathway.
